# Reliability analyses and values of isometric shoulder flexion and trunk extension strengths stratified by body mass index

**DOI:** 10.1371/journal.pone.0219090

**Published:** 2019-07-01

**Authors:** Lora A. Cavuoto, Mojdeh Pajoutan, Ranjana K. Mehta

**Affiliations:** 1 Industrial and Systems Engineering, University at Buffalo, Buffalo, New York, United States of America; 2 Industrial and Systems Engineering, Texas A&M University, College Station, Texas, United States of America; São Paulo State University (UNESP), BRAZIL

## Abstract

The main goal of this study was to investigate the reliability of muscle strength across different levels of obesity. A sample of 142 healthy subjects performed maximum voluntary isometric contractions for shoulder flexion and trunk extension on each of four days. Subjects were recruited into one of three groups, non-obese, overweight, or obese, based on body mass index (BMI). Reliability of the strength measurements within each session and across the four sessions was determined from the intraclass correlation coefficient, coefficient of repeatability, coefficient of variation, and standard error of measurement. For the shoulder flexion measures, the coefficient of variation was < 10% and intraclass correlation coefficient was > 0.75. The absolute reliability of trunk extension strength measurement was rejected due to a high variability across sessions. For both tasks, comparable strengths across the BMI groups were found.

## Introduction

A shift in the adult population to include ~1.9 billion obese (body mass index (BMI) ≥ 30 kg/m^2^) and overweight (25 ≤ BMI < 30 kg/m^2^) adults worldwide has had the negative consequence of increased risk of injuries with obesity [1]. Increasing BMI has been shown to be associated with impaired back extensor muscle function [2, 3] and shoulder pain complaints [4] during physical resistance tests. In a meta-analysis of 33 publications in the Medline and Embase databases, overweight and obesity have been linked with chronic low back pain [5]. Sedentary lifestyle and lower physical activity among individuals who are obese may contribute to weaker muscle strength. However, this negative effect can be counterbalanced with a favorable chronic weight-bearing effect as a result of increased body mass [6].

Mixed findings have been reported in the literature on the effect of obesity on muscle strength. Most studies have reported a higher absolute isometric strength of the trunk and lower extremity muscles [7–11], as well as upper extremity muscles [7, 12–16] with obesity. Interestingly, some of these studies have also reported conflicting results. For example, contrary to findings reported by Hulens et al. [8] and Rolland et al. [9], Kitagawa and Miyashita [16] reported no strength differences of the trunk and knee muscles. This increases concerns about sources of variability such as experimental protocol, equipment settings and calibration, postures, experimenters, and subjects. To guard against various sources of errors, reporting the reliability or repeatability of the strength measurements is critical [17], which has been neglected in previous studies of strength differences with obesity.

Interpretability of strength results in product and workstation design, rehabilitation decision-making, and ergonomics practice depends on the reliability of the measurements, particularly when participants with a wider range of BMI are included, as this increases the between-subject variability. Within-subject variability, such as biological changes due to fatigue, either physically or mentally, and learning effect, could also affect the results [18], and this effect can vary between subject groups. For example, lack of motivation, mental fatigue, or impaired central activation for maximal muscle contractions have been reported to be more apparent after sustained isometric contractions to exhaustion for individuals who are obese [19, 20]. Therefore, a reasonable number of subjects and trials is needed to have a higher inter- and intra-individual reliability of strength measures [18].

During repeated measurements, relative reliability reflects the degree of maintaining each individual’s rank relative to a sample of subjects, and absolute reliability shows the amount of within-subject variability [21, 22]. As suggested by Bland and Altman [23] and Rankin and Stokes [24], as no single measure sufficiently covers both relative and absolute reliability, multiple reliability metrics are needed for accurate assessment. In this study, maximum voluntary isometric contractions (MVCs) were repeated across four sessions, each on a different day, in a large heterogeneous (wide range of BMI) sample of subjects, which enabled measuring the relative and absolute reliabilities. Verifying the reliability in this study enables generalization of the findings more reliably to other conditions and individuals. Therefore, the main objective of this study was to test whether maximum muscle strength is reliable across repeated measurements across multiple sessions and across different levels of obesity. Two tasks of shoulder flexion and trunk extension were selected for the purpose of strength measurement. These muscle groups were selected for the high frequency of shoulder use during activities of daily living and the role of trunk extensor strength in supporting the more anterior center of mass, due to accumulated fat tissues around the abdomen, for individuals who are obese. Secondary objectives were to capture strength differences across subject groups and to identify significant predictors of strength. It was hypothesized that strength is affected by obesity (classified either using BMI or percent body fat). It was also hypothesized that trunk extensor strength would exhibit a greater influence of obesity due to the greater accumulation of fat tissue in the abdominal area.

## Methods

### Subjects and ethical approval

As part of a larger study on functional capacity changes related to obesity, 142 healthy subjects were recruited. Participants were assigned to three groups: 49 (24 males, 25 females) normal weight (18 ≤ BMI < 25 kg/m^2^), 50 (25 males, 25 females) overweight (25 ≤ BMI < 30 kg/m^2^) and 43 (22 males, 21 females) obese (BMI ≥ 30 kg/m^2^). Based on an acceptable reliability (*p*_*0*_) of 0.6 and an expected reliability (*p*) of 0.8, with α = 0.05, power = 0.8, and number of observations = 4, the target sample size per group was 22. All participants completed demographic, health history, and physical activity questionnaires prior to the experiment. Only healthy individuals who did not perform extensive physical activity more than one hour per day up to three days per week were included in this experiment. Detailed demographic and anthropometric information for the participants, divided by group, is provided in Table 1. As described in Mehta and Cavuoto [25], participants’ heights were measured with a standard stadiometer to the nearest 0.1 cm and weights with a digital metric scale to the nearest 0.1 kg. Waist and hip circumferences were measured to the nearest 0.1 cm using a standard flexible, inelastic measuring tape. Body fat percentage (%BF) was measured with an electronic impedance scale (TANITA Corporation, Tokyo, Japan). All study protocols were approved by the University at Buffalo and Texas A&M University Institutional Review Boards and all participants provided informed consent prior to participation. The individual pictured in this manuscript was a research assistant who recreated the postures adopted and has given written informed consent (as outlined in PLOS consent form) to publish this image.

**Table 1:** Participants’ information presented as mean (SD) by obesity level and gender.

	Normal weight		Overweight		Obese	
	Female (n = 25)	Male (n = 24)	Female (n = 25)	Male (n = 25)	Female (n = 20)	Male (n = 22)
Age (yr)*	33.8 (10.3)	28.9 (5.2)	35.8 (10.8)	31.0 (8.1)	32.0 (10.7)	30.9 (8.3)
**Body mass (kg)**^**+**^	59.4 (7.1)^A^	70.4 (5.6)^A^	71.2 (5.5)^A^	82.1 (6.4)^A^	93.3 (12.7)^B^	106.2 (15.6)^B^
Stature (cm)*	163.5 (5.1)	175.4 (6.7)	162.1 (5.0)	174.2 (6.6)	165.3 (7.0)	173.7 (5.5)
**BMI (kg/m**^**2**^**)**^**+**^	22.2 (2.2)^A^	22.9 (1.9)^A^	27.1 (1.5)^B^	27.0 (1.3)^B^	34.0 (3.1)^C^	35.1 (3.9)^C^
Body fat (%)*^,^^**+**^	28.6 (5.6)^A^	14.5 (4.5)^A^	36.4 (2.9)^B^	23.4 (4.8)^B^	42.8 (3.6)^C^	30.3 (4.0)^C^
Fat free mass (kg)*^,^^**+**^	42.1 (3.6)^A^	60.1 (5.0)^A^	45.3 (3.4)^A^	62.8 (5.6)^A^	53.0 (5.5)^B^	73.7 (9.6)^B^
Waist circumference (cm)*^,^^**+**^	75.7 (12.4)^A^	84.1 (6.2)^A^	88.5 (8.1)^B^	93.0 (6.9)^B^	102.7 (8.9)^C^	113.1 (12.6)^C^
**Hip circumference (cm)**^**+**^	93.2 (16.2)^A^	97.5 (6.3)^A^	102.5 (7.9)^B^	103.9 (7.0)^B^	115.7 (11.1)^C^	121.0 (14.8)^C^
Waist-to-hip ratio*^,^^**+**^	0.82 (0.07)^A^	0.86 (0.04)^A^	0.87 (0.07)^B^	0.90 (0.07)^B^	0.89 (0.05)^C^	0.94 (0.05)^C^

* Indicates a significant difference by gender (*p* < 0.05)

^+^ Indicates a significant difference by obesity level (*p* < 0.05)

^A,B,C^ Those that do not share a letter are significantly different at *p* < 0.05 based on a post-hoc *t*-test

### Experimental setup and protocol

Three MVCs each of shoulder flexion and trunk extension were repeated across four sessions. Sessions were separated by at least 48 hours to minimize the effect of any residual fatigue on performance, and task order was counterbalanced across subjects. The timing of sessions was based on participant availability, and thus could not be controlled across participants. Effort was made to schedule each participant at the same time of day for the four sessions, but this was not controlled. To reduce the sources of variability related to lack of motivation and inability to fully activate motor neurons, the MVCs were repeated three times and the maximum of them was selected as a measure of muscle strength for each subject in each session. Reproducibility or repeatability of the observed values was tested across sessions. In each session, participants warmed up before each task with repeated shoulder and trunk flexion, extension, and rotation. The three isometric MVCs, each 4–5 seconds long with two minutes of rest in between, were then performed, based on standard strength testing procedures [26]. Real-time visual feedback and verbal encouragement were provided during the trials. Shoulder flexion, and trunk extension were separated by at least 10 minutes each.

**Fig 1:**
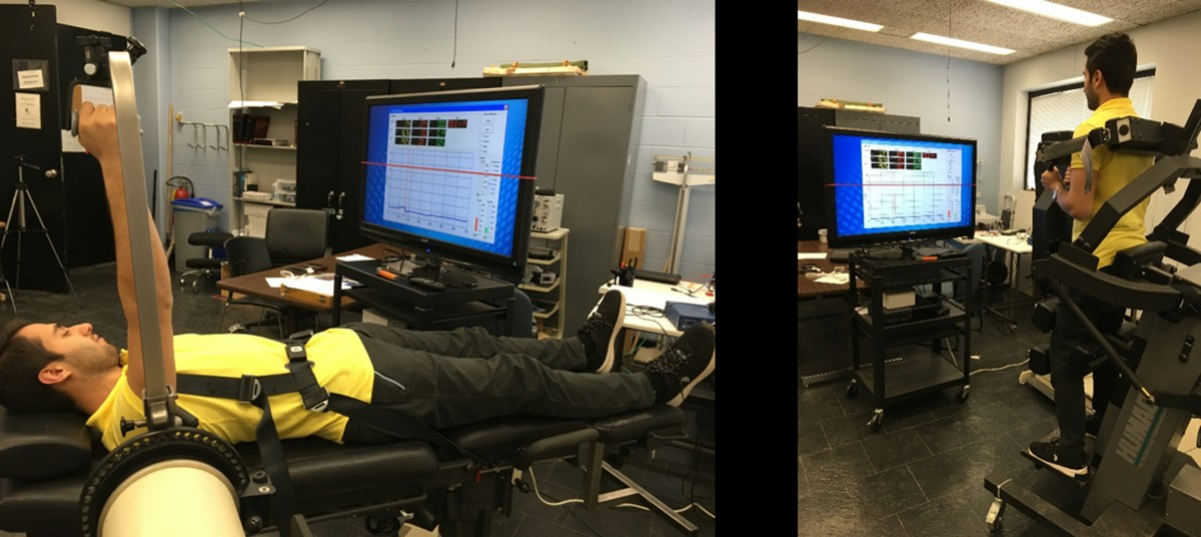
Postures used for isometric strength testing of (a) shoulder flexion and (b) trunk extension.

An isokinetic dynamometer (Cybex Humac NORM, Ronkonkoma, NY, USA) was used to measure the shoulder flexion and trunk extension torque at a rate of 100 Hz. Shoulder flexion of the right arm was tested with participants laying supine on the dynamometer chair with a seat belt around the pelvis and arm flexed at 90° with extended elbow (Fig 1A). The dynamometer’s axis of rotation and shoulder adaptor height were set with respect to the acromion process and arm length, respectively. The forearm was kept parallel to the shoulder adaptor grasping a handle with the wrist in a neutral position. Participants were allowed to release the handle during the rest periods. For trunk extension, participants stood upright on the dynamometer footplate with slightly flexed (< 5°) trunk against the sacral pad (Fig 1B). The dynamometer’s axis of rotation was aligned based on the iliac crest and L5/S1 location. The scapular and chest pads were fastened in parallel across the center of the scapulae and against the subject’s chest. The feet were placed in a fixed position against footplate heel cups separated at about shoulder width. The thigh pad, tibial pad, and pelvic belt was attached to help firmly securing the lower body minimizing the confounding effect of other muscles during trunk extension.

### Measures and statistical analysis

Due to differences in strength by gender, and a hypothesized interaction between obesity level and gender on strength, all analyses were conducted separately for males and females. For each task, the level of absolute agreement across the four MVCs for each person was evaluated using the intraclass correlation coefficient (ICC). ICC estimates and their 95% confidence intervals were calculated based on a single measure (k = 1), absolute agreement, two-way random effects model. K = 1 was chosen since strength testing is typically performed on a single day with the intent of using that value as the representative strength for an individual. The ICCs, as a measure of relative reliability, were calculated for the overall sample of subjects as well as for each of the three groups separately to evaluate between-group differences in reliability [27, 28]. A high agreement (ICC > 0.9) indicates excellent reliability of repeated MVCs as a measure of strength for each person within each task [21]. The interpretation for the ICC was based on the benchmarks suggested by Landis and Koch [29] and updated by Shrout [30]: values between .4 and .6 were considered fair, .61-.8 moderate, and .81–1 substantial. Since the ICC is a relative measure of reliability and cannot be used to assess trial-to-trial differences, additional absolute indices of reliability were calculated [22, 27], including the coefficient of repeatability (CR), standard error of measurement (SEM), and coefficient of variation (CV) with its 95% confidence interval [17, 31]. CR, suggested by Bland and Altman [23] for the case of repeated measures, was calculated as 2.77*s*_*w*_, where *s*_*w*_ is the within-subject standard deviation from the one-way analysis of variance (ANOVA) with subject as the factor. The mean CV was calculated as the average of the within-subject standard deviations divided by the within-subject means. Variability less than 10% of the mean (CV < 10%) indicates that about 68% of the measurements are within 10% of the mean [32]. SEM was calculated overall and by group using the relevant ICC and standard deviation of the sample strength measures ($SEM=SD\sqrt{1-ICC}$). SEM eliminates the effect of inter-individual variability calculated in ICC [17]. The SEMs were then used to provide 95% CIs for the true strength measurements quantified as strength ± 1.96(SEM) for each task and overall population. In addition, minimum detectable change (MDC) was calculated as SEM×1.96×√2 to show the minimum considerable real change in the performance [27].

Linear regression analysis was performed for each task in order to find the linear trend of strength changes with age and either increasing BMI or percent body fat (%BF). The strength data was then used to provide the percentile values of strength for each task overall as well as for each group. The variability of the strength in the commonly used submaximal percentiles (i.e., 5^th^, 10^th^, 25^th^, etc) in workstation, task, and product design was also considered. All statistical analyses were conducted in SPSS Ver. 24 (IBM Corporation) and the level of significance was set at α = 0.05.

## Results

### Reliability of strength measurements

The ICC and CV with 95% CIs, CR, and SEM results by gender, overall and by BMI group, are presented in Table 2. Overall strength measurement across sessions had substantial reliability as indicated by the ICC values exceeding 0.8 for both genders and tasks. If the average measure was used, these ICCs would be expected to increase. When divided by BMI group, reliability remained good, with all group-level ICCs > 0.7. The lowest mean ICC was for the obese female group for trunk extension. The lower bound for the 95% confidence interval was above 0.6 in all but one case (obese female for trunk extension). Therefore, MVC measurement from a single day can be used reliably for further analysis. Variability about the mean among the replications ranged from 1.24–26.42 for the shoulder flexion and 2.54–43.30 for the trunk extension task. Measures of strength for the shoulder flexion task were the most reliable, which is evident from the lower SEM and CR values. Generally, higher variability was observed for the trunk extension compared to the shoulder flexion task, and for males compared to females. For females, the normal weight group had lower variability between the tasks compared to the overweight and obese groups across all measures, with the overweight group having the largest CR value and the obese group having the largest SEM and CV values. For males, the pattern of the overweight group having the largest CR remained for both tasks. The overweight male group also had larger CV and SEM values for the trunk extension task, but not for shoulder flexion.

**Table 2:** ICC and CV with 95% CI, CR, and SEM results.

	ICC	CR	CV (%)	SEM
**FEMALE**				
**Shoulder Flexion**				
Overall	.816 (.748,.872)	10.47	9.15 (7.86,10.45)	1.87
Normal	.881 (.796,.939)	7.73	8.51 (6.32,10.70)	1.57
Overweight	.768 (.626,.878)	13.59	9.42 (7.23,11.61)	2.05
Obese	.786 (.632,.899)	8.34	9.62 (7.17,12.07)	2.03
**Trunk Extension**				
Overall	.838 (.777,.889)	41.93	15.44 (13.52,17.36)	7.73
Normal	.897 (.823,.948)	29.44	11.96 (8.91,15.01)	6.05
Overweight	.853 (.741,.927)	54.09	16.15 (13.10,19.20)	7.98
Obese	.742 (.565,.876)	38.43	18.89 (15.48,22.30)	8.30
**MALE**				
**Shoulder Flexion**					
Overall	.833 (.771,.884)	17.16	8.72 (7.63,9.80)	3.04
Normal	.866 (.769,.934)	15.15	8.65 (6.76,10.55)	2.90
Overweight	.814 (.694,.903)	17.48	8.69 (6.84,10.55)	3.04
Obese	.812 (.681,.906)	15.98	8.82 (6.84,10.80)	3.17
**Trunk Extension**					
Overall	.813 (.744,.870)	74.77	15.88 (13.77,17.99)	13.36
Normal	.841 (.726,.921)	49.54	12.78 (9.23,16.34)	10.84
Overweight	.797 (.670,.893)	100.83	18.34 (14.86,21.83)	14.99
Obese	.825 (.697,.915)	68.52	16.47 (12.75,20.18)	13.46

Additionally, mean, 95% CI of the true strength estimates, and MDC for the overall sample of subjects by gender and for each task are presented in Table 3. MDCs for females are 15.1% and 25.6% of the mean for shoulder flexion and trunk extension tasks, respectively. For males they are 14.3% and 26.9%, respectively.

**Table 3:** 95% CI for true estimates of strength values.

**FEMALE**			
**Task**	**Mean**	**95% CI**	**MDC**
**Shoulder Flexion (Nm)**	34.47	(30.80, 38.14)	5.19
**Trunk Extension (Nm)**	83.57	(68.43, 98.72)	21.42
**MALE**			
**Task**	**Mean**	**95% CI**	**MDC**
**Shoulder Flexion (Nm)**	58.86	(52.90, 64.81)	8.43
**Trunk Extension (Nm)**	137.63	(111.44, 163.81)	37.03

### Effect of obesity on strength

**Fig 2:**
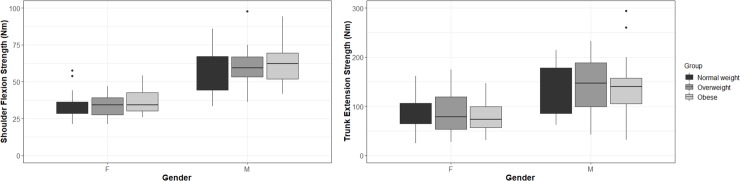
Boxplots of the strength outcomes and the variability across participants.

Regression analyses revealed no significant associations between BMI and strength or body fat percentage and strength for shoulder flexion and trunk extension strengths. Age was not significant in the regression models either. For practical purposes, strength percentiles of each task, overall and for each obesity group, are provided in Table 4. Fig 2 presents boxplots of the strength outcomes and the variability across participants.

**Table 4:** Strength distributions for shoulder flexion and trunk extension.

	Overall	Normal weight	Overweight	Obese
**FEMALE**				
**Shoulder Flexion (Nm)**				
5	22	21	22	26
10	23	24	23	27
25	28	28	27	30
50	34	32	34	34
75	39	37	40	44
90	45	48	44	47
95	50	56	46	54
**Trunk Extension (Nm)**				
5	31	27	29	31
10	35	35	32	38
25	56	64	50	57
50	77	80	79	73
75	107	108	121	101
90	137	151	134	136
95	152	160	164	147
**MALE**				
**Shoulder Flexion (Nm)**				
5	40	35	37	42
10	43	41	40	43
25	46	44	53	51
50	59	50	59	62
75	68	70	67	70
90	78	80	73	79
95	82	84	91	92
**Trunk Extension (Nm)**				
5	46	62	43	38
10	62	62	46	69
25	91	77	97	98
50	146	152	147	140
75	178	178	195	161
90	216	201	228	242
95	232	213	232	289

## Discussion

### Reliability of strength measurements

Absolute and relative reliabilities of the shoulder flexion strength measures were supported by having agreement between the measurements ICCs > 0.75, variabilities about the means < 10%, and repeatability coefficients < 15 for females and < 20 for males [31, 32]. These results are consistent with those for healthy young adults, including those for isometric elbow flexion strength [33], isokinetic shoulder peak torque (21), and isokinetic trunk flexion [34]. For the trunk extension task, while relative reliability remained substantial for most groups (only two had ICC < 0.8), the absolute reliability of the measures was not shown (CV% > 10% for all groups and CR > 25 with large differences between groups). For this task, in both females and males, the normal weight group had a lower mean CV (12.0% and 12.8%, respectively) compared to the overweight (16.2% and 18.3%) and obese groups (18.9% and 16.5%). Similarly, the normal weight group had a lower CR compared to the overweight and obese groups, with the overweight group having the largest coefficient of repeatability. The heterogeneous sample over a large BMI range resulted in an increased coefficient of variation for the trunk strength compared to shoulder strength. Larger variability during trunk exertions was previously reported and attributed to the complex muscle involvements and co-contractions involved [35]. In addition, while the experimental setup was designed to minimize recruitment of supporting muscles, such as the quadriceps, by controlling the posture and limiting movement (as has been done in other studies [34]), participants may adjusted their posture during the task and used their legs in generating the trunk extension movement. Measuring trunk extensor strength in other postures or settings, such as a seated posture, might be required to increase the stability of the muscles involved and minimize unintended activation of antagonist muscles, to increase the reliability and precision of the measures.

Findings of this study suggest that individual changes in strength equal or greater than ~15% and ~26% of the mean above or below the previous score can be interpreted as a real change, with 95% confidence, for shoulder flexion and trunk extension tasks, respectively. These MDCs help in performance assessments and return to work evaluations to test the effect of an intervention on performance improvements [27].

### Effect of obesity on strength

The present study found comparable trunk extension strength between groups of individuals who are obese and non-obese; a finding consistent with previous studies [13, 16]. However, the former classified obesity based on %BF rather than BMI, and overweight individuals were not considered in either of the studies. In contrast, Hulens et al. [8] found significantly higher absolute trunk extension strength, but these results may be attributable to a sample that included individuals who were extremely obese and/or older, along with a lack of control over participant physical activity. As described by Pajoutan et al. [36], trunk strength has previously been shown to be positively correlated with fat-free mass. When obesity-related increases in strength have been found for the lower extremity or trunk (e.g., Maffiuletti et al. [10] and Lafortuna et al.), chronic training and increased cross-sectional area of the muscle have been hypothesized. While larger cross-sectional area has been found with obesity [37], the muscle contraction can be impaired by the presence of intramuscular fat [38].

Contrary to the present study findings, two other studies [12, 13] found significantly higher shoulder flexion strength with obesity, with ~20–25% higher strength for the obese groups in those studies. Mean shoulder flexion strength in both of those studies was ~50 Nm for the non-obese groups and ~60–65 Nm for the obese groups, using a similar posture to the one adopted in the current study. While there was not a significant difference in shoulder flexion strength in the current study, these means are similar to those observed for the current male sample, where the 50^th^ percentile strengths were 50 Nm for the normal weight group and 62 Nm for the obese group. The prior studies do not distinguish strength outcomes by gender, but differences in the sample and participant physical activity levels may explain the lower female strength values in the current study compared to previously reported overall means.

Comparable strength across obesity levels aligns with findings reported in existing studies of hand grip strength as well [8, 9, 35]. For example, in a large study of 1443 elderly subjects in all three BMI categories, isometric grip strength remained intact with obesity [9]. In that study, while obesity was not a significant factor, recreational physical activity was found to be the determinant of the handgrip force capacity. Since the present study sample was similar in their physical activity levels, i.e., recreationally active to sedentary individuals, we were unable to test strength predictability based on physical activity levels. It is recommended that future studies address this by including subjects across different physical activity levels.

Age was not a significant negative linear predictor of strength in any of the regression models. This is not contrary to expectation, as most studies that have identified a significant age effect have considered samples with individuals greater than 60 years old, versus 56 in the current study. For example, Eksiolgu [39] observed equivalent hand grip strength for males 18–59 and the only significant differences for females were for those greater than 50 years old. Similarly, in a meta-analysis of reference values for adult hand grip [40], the age-related decline in grip strength (for either hand) occurs from the 55–59 years group and older. While muscle strength values have shown to differ by ethnicity/race groups Blakley et al. [41], these differences have shown to be influenced by factors such as age [42]. Given that obesity rates are higher in African American and Hispanic adults in the United States [43], research is warranted to document strength values stratified by race/ethnicity, obesity, and age groups from to represent current workforce demographics.

### Limitations

While the reliability of the measures was verified for the shoulder flexion task in a large and heterogeneous sample of subjects, representative of the population, it is worth mentioning that the results of this study can only be extended to younger healthy adults, for the specific tasks tested. The BMI and age recruitment criteria for this study were limited to Class I and II obesity (30 < BMI < 40 kg/m^2^) and younger adults. Extremely obese individuals were excluded since they represent ~6% of the population [44]. While efforts were made to recruit workers in the present study, the study sample largely comprised of university students and residents from local communities. In addition, experimental sessions were scheduled based on participant availability and thus the time of day was not controlled. Despite potential circadian influence, reliability remained high. Future work should control timing of strength measurements to confirm these findings. The study also only considered the reliability of isometric strength measurements. Future studies should consider dynamic strength tasks where the influence of body mass support may have a larger impact on repeatability of strength measurement. Generalizing the results to other postures, tasks, populations, and individual factors requires further research.

## Conclusion

Across obesity levels, strength measurements had high levels of absolute and relative reliabilities for the shoulder flexion task, even when accounting for various sources of errors (e.g., repeated measurements). The findings of this study for the shoulder flexion task support the use of a single strength measurement for practical purposes to estimate the required strength and allowances in product design and performance evaluation. Trunk extension strength values should be used with caution. While the relative reliability was good to substantial, the absolute reliability measures showed high variability across sessions when considering gender and BMI group.
